# A Systematic Comparison of High-End and Low-Cost EEG Amplifiers for Concealed, Around-the-Ear EEG Recordings

**DOI:** 10.3390/s23094559

**Published:** 2023-05-08

**Authors:** Michael Thomas Knierim, Martin Georg Bleichner, Pierluigi Reali

**Affiliations:** 1 Institute of Information Systems & Marketing, Karlsruhe Institute of Technology, 76131 Karlsruhe, Germany; 2 Neurophysiology of Everyday Life Group, Department of Psychology, University of Oldenburg, 26129 Oldenburg, Germany; martin.georg.bleichner@uol.de; 3 Research Center for Neurosensory Science, University of Oldenburg, 26129 Oldenburg, Germany; 4 Department of Electronics Information and Bioengineering, Politecnico di Milano, 20133 Milan, Italy; pierluigi.reali@polimi.it

**Keywords:** concealed EEG, cEEGrids, Auditory ERP, timing test, Smarting Mobi, OpenBCI

## Abstract

Wearable electroencephalography (EEG) has the potential to improve everyday life through brain–computer interfaces (BCI) for applications such as sleep improvement, adaptive hearing aids, or thought-based digital device control. To make these innovations more practical for everyday use, researchers are looking to miniaturized, concealed EEG systems that can still collect neural activity precisely. For example, researchers are using flexible EEG electrode arrays that can be attached around the ear (cEEGrids) to study neural activations in everyday life situations. However, the use of such concealed EEG approaches is limited by measurement challenges such as reduced signal amplitudes and high recording system costs. In this article, we compare the performance of a lower-cost open-source amplification system, the OpenBCI Cyton+Daisy boards, with a benchmark amplifier, the MBrainTrain Smarting Mobi. Our results show that the OpenBCI system is a viable alternative for concealed EEG research, with highly similar noise performance, but slightly lower timing precision. This system can be a great option for researchers with a smaller budget and can, therefore, contribute significantly to advancing concealed EEG research.

## 1. Introduction

Wearable electroencephalography (EEG) offers promising application potentials for brain–computer interfaces (BCI) to improve everyday life, for example, in the form of sleep improvement [1], seizure detection [2], adaptive hearing aids [3], or thought-based control of digital or robotic devices [4,5]. To realize these innovations, scholars are increasingly turning to miniaturized, and less visible EEG systems (so-called transparent, or concealed EEG) that are still capable of reliably capturing neural activity at the millisecond scale [6], and that approach usability in everyday life situations [7,8,9]. These researchers use custom-made in- or around-the-ear EEG systems [2,9], or, alternatively, ready-to-use flex-printed EEG electrode arrays that can be attached around the ear (“cEEGrids” [10]). Thereby, they collect high quality EEG signals related to auditory attention, for example to study whether they can detect attentiveness to sounds in everyday situations [7,8,11]. By using these ear-EEG solutions, researchers can overcome some of the major limitations of traditional cap-based EEG, in particular the cumbersome setup procedures (which include repeated hair washing) and the unpleasant aesthetics (who wants to wear a swim cap filled with gel and connected to 32–64 electrodes in everyday life?).

However, while these concealed EEG approaches represent an intriguing development, their use is somewhat limited due to the inherent measurement challenges. As the electrode space is reduced (compared to conventional EEG), the recorded signal amplitudes are also reduced (the effects of interest are on the order of a few microvolts) [3]. Therefore, the amplifiers used need to show high recording sensitivity to enable data collection with these miniaturized EEG electrode designs. As a result, the amplification systems used to date have mostly been high-end products with high costs [12]. This high cost, in turn, is a major limitation to the widespread adoption of concealed EEG designs. For this reason, previous research has, for instance, attempted to study whether around-the-ear cEEGrid electrodes can also be used with lower-cost open-source amplification systems, such as the OpenBCI Cyton+Daisy boards [12]. The authors of [12] found a general suitability of the OpenBCI amplifiers to collect large-effect neural activity changes in the frequency domain, such as the increase in occipital Alpha frequency power during eye closure (the Berger effect [13]), or a decrease in Alpha band power with increasing task difficulty [12] which were in line with previous work using high-end amplifiers [10,14]. However, these authors did not directly compare the performance of the OpenBCI amplifiers with that of a high-end amplifier, nor did they evaluate the aforementioned critical performance factors (timing accuracy and signal-to-noise ratio—SNR), which are essential for several applications (e.g., auditory evoked potential detection). Furthermore, previous research has also reported low input-referred noise (~1 µVpp) and low power consumption (5 mW/channel) for the OpenBCI Cyton+Daisy [15]. In addition, this OpenBCI amplifier has shown a high SNR compared to clinical-grade amplifiers [16] and the usability of this amplifier for event-related potential (ERP) research with classical cap-EEG [15,16]. Nevertheless, it remains to be investigated whether the system can be reliably used for the more challenging recording situation with concealed EEG designs.

In this article, we provide a thorough comparison of the Cyton+Daisy boards (OpenBCI, New York, NY, USA) with a benchmark amplifier, the Smarting Mobi (MBrainTrain, Belgrade, Serbia). To this end, we conducted three studies to assess the temporal accuracy of both systems, their performance in a typical laboratory study design, and finally a direct comparison of signal quality through simultaneous data recording with both amplifiers on the same participant. In doing so, we focus on concealed EEG recordings with the around-the-ear cEEGrid electrodes because they are readily available and don’t require custom fabrication for individual subjects. Initially, with the default settings of both systems, we found large differences in temporal precision, with high timing variation in the OpenBCI Cyton+Daisy, which would greatly reduce the ability to record time-domain features (ERPs). However, by correcting the Bluetooth dongle buffer settings and, most importantly, by developing a timestamp correction algorithm (provided with the article), the temporal precision of this low-cost amplifier was significantly improved, approaching the precision of the Smarting Mobi. In addition to the temporal accuracy, frequency and time domain comparisons of the amplifiers in the two recording setups with human participants show highly comparable recordings. This confirms the applicability of the OpenBCI system for these challenging EEG recordings, a finding that we believe provides a valuable foundation for further advancing research on concealed EEG.

## 2. Materials and Methods

**Figure 1:**
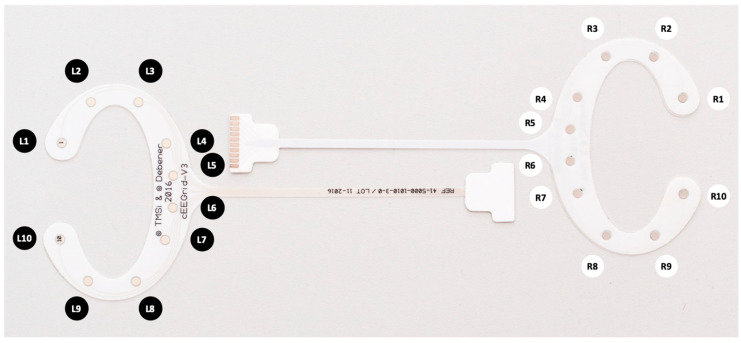
Two cEEGrid electrodes showing the electrode positions for left and right ear.

As reference for a concealed EEG recording method, we use the so-called cEEGrids—a ‘flexible printed Ag/AgCl electrode system consisting of ten electrodes arranged in a c-shape to fit around the ear’ [10] (see Figure 1) in this work. While they are not the only available option for concealed EEG (see e.g., [9,17,18] for in-ear EEG approaches), we focus on the around-ear method as the electrodes are readily available, can be used without personalization or customization, and come with multiple electrodes which allows recording signals across a range of positions on the head. Because of these multiple positions, the cEEGrids allow characterizing known EEG phenomena by providing additional information (e.g., the strength of an effect or feature morphologies on different positions around the ear [3]). At the same time, it should be highlighted, that these cEEGrids—much like other ear-EEG solutions—only capture a subset of neural information when compared to traditional cap EEG [3]. Specifically, they capture sources close to the ear region well (e.g., from the auditory cortex [6,19,20]). More distant sources (e.g., from anterior or central brain regions) are harder to observe [3]. The cEEGrids have been repeatedly reported to enable comfortable, high-quality and multiple-hour EEG recordings in field settings [7,8,10,11]. The recording quality is primarily realized by the possibility of using the cEEGrids with a gel enclosed by the adhesive [10]. This property is also essential for our signal quality comparisons as dry electrodes are typically much more prone to irregular recording artefacts [21]. The electrodes’ application around the ear is realized in about five minutes (including light cleaning of the skin with alcohol or an abrasive gel) [22]. The electrodes can be re-used numerous times after cleaning the gel residue and re-applying a double-sided adhesive. For thorough application instructions we refer the reader to [22].

**Figure 2:**
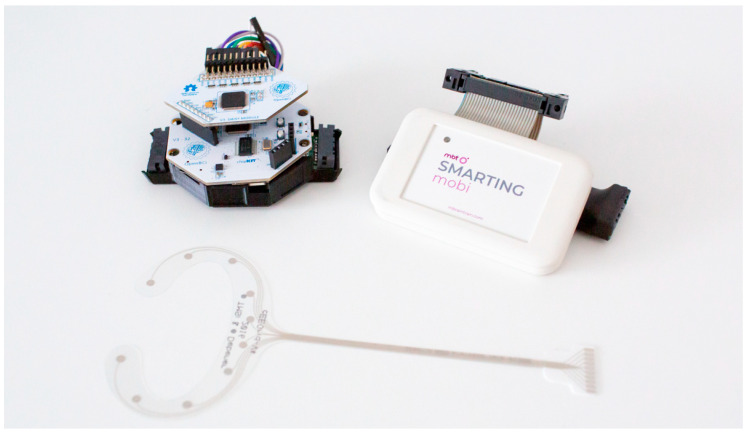
OpenBCI Cyton+Daisy (on the **left**—the board sitting on top is the Daisy extension) and MBrainTrain Smarting Mobi (on the **right**). A cEEGrid electrode is shown **below**.

The cEEGrids are then connected to the two amplifiers compared in this study: the OpenBCI Cyton+Daisy, and the MBrainTrain Smarting Mobi 24 (see Figure 2). The OpenBCI amplifier comes in two configurations: either as the standalone Cyton amplifier (with 8 recording channels) or extended to the Cyton+Daisy configuration with 16 recording channels. In the eight-channel configuration, EEG data can be recorded with a sampling frequency of 250 Hz. This temporal resolution is reduced to 125 Hz in the 16-channel version due to limitations in the wireless packet transmission bandwidth. It is possible to also collect data with 250 Hz for the Cyton+Daisy configuration when data is not directly streamed to a recording computer, but instead stored on an SD card. Data from the Cyton+Daisy can be streamed to a computer using an RFDuino Bluetooth 4.0 Low Energy (BLE) radio transceiver. Due to low power consumption and the option to pair the Cyton+Daisy with battery sizes up to 1000 mAh, this amplifier system allows continuous recordings for over 12 h, which enables full-day data collections (e.g., to conveniently monitor neural activity in field study settings that span an entire day). Importantly, all of the OpenBCI components (hardware and software) are open-source and the amplifiers come with a much lower price than high-end systems like the Smarting Mobi. For the higher price, the Smarting Mobi features certain advantages like a higher sampling frequency of 500 Hz, up to 22 recording electrodes, and very low input-referred noise (<1 μVpp). Data from the Smarting Mobi can be streamed to a computer or portable device using a BlueSoleil Bluetooth Dongle Class I (Type BS002) with the Bluetooth v2.1 + EDR transmission protocol. Furthermore, in contrast to the OpenBCI amplifiers, the Smarting Mobi uses an active ground electrode (driven right leg—DRL) configuration. Other than that, the two amplifiers share many similarities like an amplification gain of up to factor 24, and a resolution of 24 bits. Additionally, both amplifiers are fitted with three-axis accelerometer sensors to observe head movement during recordings. Table 1 summarizes the features of both amplifiers.

While these specifications are available from the respective user manuals, we wanted to put both systems to the test in a challenging EEG recording scenario: concealed ear-EEG where high temporal precision and low noise are essential. Therefore, we pursued three studies that are reported below. All studies used the same technical setup (i.e., the same hardware/software combination). As main recording device, a Microsoft Surface Laptop 3 was used running Windows 10 Home (Version 10.0.19042), NeuroBS Presentation Version 22.1 for the presentation of the audio stimuli, Smarting Streamer Version 3.4.3 for the recording with the Smarting Mobi amplifier, and the OpenBCI LSL Python interface (https://github.com/openbci-archive/OpenBCI_LSL, accessed on 10 June 2021), executed in JetBrains PyCharm 2020.2 running Python Version 3.6.0. Finally, LabRecorder Version 1.14.0 was used to collect all data streams using the LabStreamingLayer (LSL) protocol (https://github.com/sccn/labstreaminglayer, accessed on 10 June 2021).

## 3. Timing Test

As a first step, we decided to test the timing precision of the two amplifiers in a highly controlled setup. By feeding a consistent voltage-modulated signal (i.e., a square wave generated through the integrated sound card of a PC) into the amplifiers repeatedly, the actual timing precision can be assessed by having a clear ground-truth metric without any biological, or behavioral signal contaminations from human study participants. Our timing tests were closely aligned with the procedure in [23], thereby focusing on the temporal precision between the presentation of a physical stimulus and the recorded event markers.

### 3.1. Protocol

Using the EEG amplifiers as oscilloscopes, we adapted the protocol from [23] to a desktop PC, which allowed us to evaluate and quantify the temporal precision of the amplifiers using audio signals. The core part of this timing test protocol is that the signal on the audio jack is fed directly into the EEG amplifiers, whose signals are transmitted through the Bluetooth dongle and recorded by the corresponding desktop PC. This setup can measure the time between the programmatic start of the playback of a sound, marked by a stimulus event marker, and the actual playback onset of the sound, as indicated by the audio jack voltage fluctuations, with EEG sampling rate precision (here: 125 to 500 Hz sampling rate, resulting in 8 ms to 2 ms precision). To prevent possible damage to the amplifier and a clipped signal, the volume is set to a medium level (35%). Additionally, capacitors (ZSU 100 nF 20% 50 V RM2.54) were integrated in the circuit to prevent accidental power surges from damaging the amplifiers.

**Figure 3:**
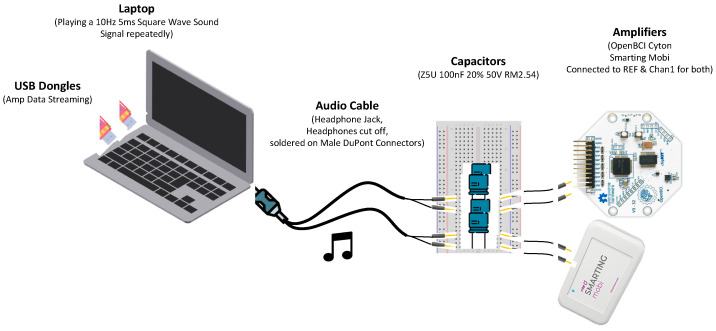
Timing test setup. The varying delay between the programmatic start of a sound playback and the actual onset is evaluated with a desktop PC running the Smarting Streamer and OpenBCI LSL Python application and the NeuroBS Presentation application. A marker sent by Presentation indicates the onset of the sound playback and is recorded by both amplifiers simultaneously alongside the voltage fluctuations fed from the audio jack into the EEG amplifier.

To record the signal, the stimulus presentation application (NeuroBS Presentation) plays a sound and sends out an LSL marker indicating the intended playback time, which is recorded in the EEG acquisition file. The sound signal is picked up from the headphone jack and is recorded on a single EEG channel using a cable connection. Both amplifiers are connected to the recording PC at the same time (see Figure 3). The signal data is transmitted to the PC using the respective, proprietary Bluetooth dongles of each device manufacturer. We used the integrated sound card of the PC to produce a periodic square wave signal (10 Hz frequency, 5 ms duration) for the timing tests. This setup allowed us to quantify the delay between the generation of each square wave and its detection by the tested EEG amplifiers. The same number of trials were presented for each recording (~400) in a single block.

Multiple meaningful configurations are possible for comparing the timing in the two amplifiers, which is why we ran the timing test in three configuration pairs.

***Configuration 1:*** First, recordings were collected using the typical recording parameters in each amplifier, as they are used in the physiological evaluation study (i.e., sampling frequencies of 500 Hz for the Smarting Mobi and 125 Hz for the 16-channel Bluetooth recording with the OpenBCI Cyton+Daisy). These data can show how the amplifiers would perform in their default configurations.

***Configuration 2:*** To more directly assess possible timing differences, we collected a set of recordings in which both amplifiers were set to the highest common sampling frequency of 250 Hz. For the Smarting Mobi, this can be set up in the Smarting Streamer Application. For the OpenBCI LSL Python interface, a higher sampling rate can be used when data is only collected using an eight-channel OpenBCI Cyton setup.

***Configuration 3:*** We learned that the regular OpenBCI Dongle configuration is considered insufficiently precise for ERP studies by the manufacturer due to the FTDI buffer latency timer being set to an inadequately long interval (16 ms) by default. Therefore, the manufacturer recommends lowering this setting to 1 ms (https://docs.openbci.com/Troubleshooting/FTDI_Fix_Windows/, Last accessed on 15 March 2023). The third set of recordings was, therefore, collected after changing this buffer setting, one set with the regular sampling frequencies (Smarting Mobi: 500 Hz and OpenBCI Cyton+Daisy: 125 Hz), and one set with the highest common sampling frequency (250 Hz).

Across all configurations, the cables connecting directly to the reference and first channel pins in each amplifier were switched after every four recordings, to eliminate possible confounding influences in the circuits. Altogether, 32 recordings were made with 400 trials per recording (eight for each configuration). The data and code for these timing tests are available at https://github.com/MKnierim/openbci-vs-smarting-timing-test.

### 3.2. Data Processing

#### 3.2.1. Regular Dejitter and Signal Peak Detection

**Figure 4:**
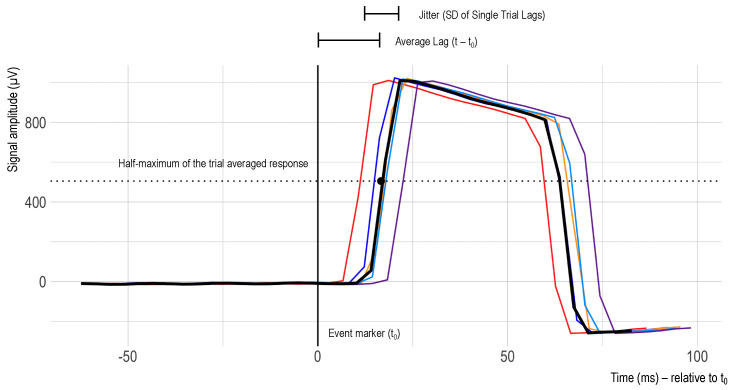
Exemplary excerpt of timing test trial recordings. The difference between the event marker (t_0_ = 0 ms) and the half-maximum of the rising edge of the square wave signal varies from trial to trial. The colors represent different trials.

To extract the single trial epochs from the continuous recording of the square wave signal, the recorded data were cut between −200 ms and +800 ms after the stimulus software marker. The timestamp of this software marker was thus set as the timing reference (t_0_). Afterwards, the delay between the stimulus marker and the signal amplitude increase (onset) was assessed. In alignment with [23], the single trial latency was defined as the time between marker onset and the amplitude exceeding the half-maximum of the trial-averaged response. Additionally, latency jitter was defined as the standard deviation of those single trial latencies, and latency lag was defined as the mean of the single trial latencies (see Figure 4).

It is important to note the inherent presence of a slight jitter in the sample time stamps as the timestamping itself usually does not happen exactly in regular intervals but on a somewhat random schedule (dictated by the perils of the hardware, drivers, and operating systems). To remove this jitter, the XDF file importer (e.g., the Python interface pyxdf which we used here—https://github.com/xdf-modules/pyxdf, accessed 10 June 2021) uses a robust linear interpolation method (i.e., adjusting the timestamps with a linear model fit in signal segments without gaps). This approach performed as expected for all recordings with single packet transmission (all Smarting Mobi recordings and the OpenBCI recordings in Configuration 3 with 1 ms FTDI buffer settings). In contrast, the observed trial latencies for the default OpenBCI recordings (with 16 ms FTDI buffer—Configuration 1 and 2) showed a highly erratic pattern. These observations led us to further investigate the suitability of alternative dejittering approaches for the OpenBCI data.

#### 3.2.2. Chunk-Dejittering

**Figure 5:**
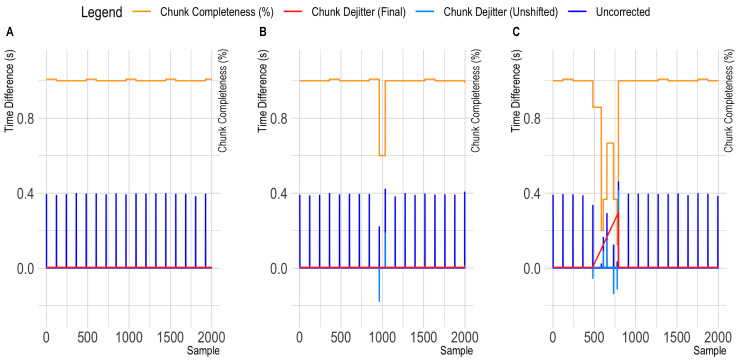
Observed patterns in OpenBCI Cyton sample-to-sample differences. The orange line highlights the variation in sample completeness (here: samples per chunk divided by 120). The dark blue line indicates the time distance between the original time stamps. The red line indicates the time difference between corrected time stamps. (**A**): Showing a clean data segment. Here, the differences between the corrected time stamps (red line) are similar to the sample-to-sample differences in all the Smarting Mobi recordings. (**B**): Showing the occurrence of a single short chunk. The negative time difference highlighted in the light blue trace indicates the overlap of the irregular chunk with the previous one after within-chunk timestamp extrapolation. (**C**): Showing the occurrence of an irregular chunk sequence.

The default buffer configuration of the OpenBCI Bluetooth dongle leads to an accumulation of data samples in packets that are then received at more or less regular intervals. Thereby, the time structure in this data resembles chunks with low sample-to-sample time difference within chunks, and large differences between the last sample and the following chunk (see Figure 5A). Importantly, slight variations can be observed in these chunk sizes (59–61 samples for 125 Hz recordings and 119–121 samples for 250 Hz recordings), which explains why the linear XDF interpolation method (regular dejitter) performed poorly with this timestamp structure.

**Figure 6:**
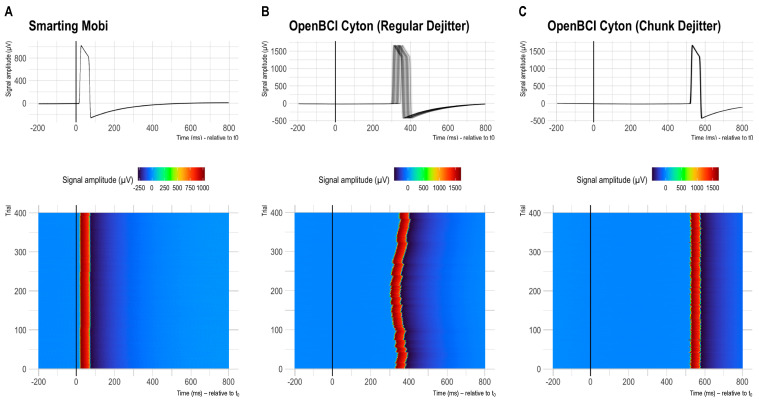
Distributions of single trial latencies (and resulting jitter) for run 12 (configuration 2—common 250 Hz sampling frequency for both amplifiers). (**A**,**B**) show the respective results with the default recording configurations. (**C**) shows the OpenBCI timing with the novel chunk-dejitter method.

Assuming good temporal accuracy for the chunk reception timestamps, we pursued an alternative dejittering approach for the OpenBCI data that focused on within-chunk (i.e., local) timestamp correction instead of a global timestamp correction. Thereby, the reception of a new chunk is identified by their large sample-to-sample time difference first, and afterwards, the timestamps for each chunk are extrapolated from the first sample (the original chunk reception timestamp) using the underlying sampling frequency. Initially, this approach showed a substantial improvement in jitter metrics, but produced outliers with some trials. Inspecting the sample-to-sample timespans again further highlighted that the previously mentioned irregular chunks now appeared to overlap with previous chunks (and with gaps to following chunks—see Figure 5B). To further correct this issue, short chunks were shifted in place (i.e., moved to the right by adding the delta value to the chunk timestamps). This process appeared to correct the timestamp information (see Figure 5C), as can also be seen in the example of a recording from Configuration 1 in Figure 6. Therefore, this chunk-dejitter algorithm was also used for analyzing the timing performance in the OpenBCI recordings in Configuration 1 and 2. To enable other researchers to utilize this dejitter method, we also integrated the algorithm in a fork of the pyxdf library that is available at: https://github.com/MKnierim/pyxdf (see Supplementary Materials).

### 3.3. Results

**Figure 7:**
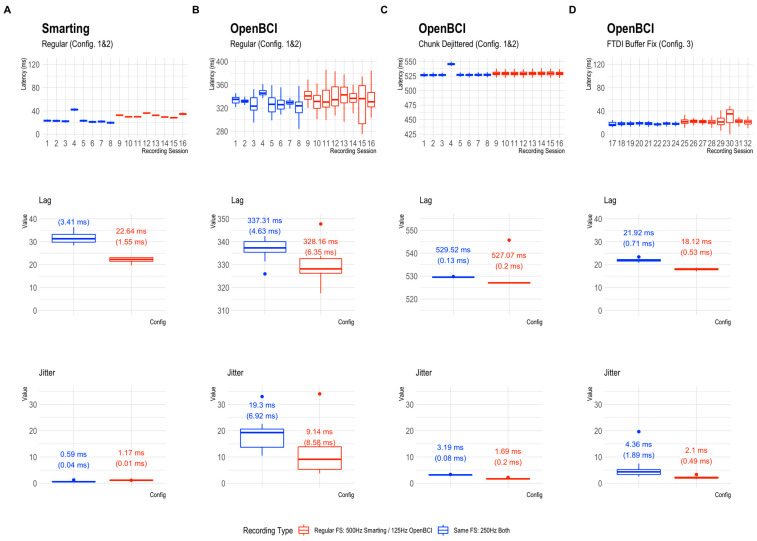
Latency distribution boxplots. (**A**): Smarting Mobi recordings. (**B**–**D**): OpenBCI Cyton+Daisy recordings in configurations 1–3. Note the different levels of the y-Axis scales. The y-Axis range is consistent (showing 150 ms) but the range is different for plots (**B**,**C**), indicating a higher overall latency in the OpenBCI recordings with the default 16 ms FTDI buffer setting. The second and third row show the corresponding lag and jitter statistics for the different configurations. The values on the boxplots show median values and IQRs as robust distribution summaries.

The global results of the timing comparisons for all 32 audio marker recordings (runs) are summarized below. The boxplots in Figure 7 reflect the onset latency distributions based on ~400 presented stimuli per session in the top row, and the average lag and jitter metrics in the mid and bottom rows, respectively. Note that the y-Axis axis range is consistent (showing 150 ms) but the level is much higher for plots B and C (OpenBCI Config 1 and 2).

Across the runs and configurations, the lag was found to be the lowest for the Smarting Mobi (31.3 ms at 500 Hz and 22.64 ms at 250 Hz), and the OpenBCI recordings with the FTDI buffer fix (21.92 ms at 125 Hz and 18.12 ms at 250 Hz). For the OpenBCI recordings with the default buffer settings, the lag was much larger both with the regular dejitter method (337.31 ms at 125 Hz and 328.16 ms at 250 Hz) and the chunk dejitter method (529.52 ms at 125 Hz and 527.07 ms at 250 Hz). This observation implies that the observation of a classical ERP component (e.g., the P300) would be critically skewed in this default buffer configuration, if the lag is not accounted for. This problem is exacerbated by a substantial variation in lag per run that is present with the regular dejitter method. In contrast, the chunk dejitter method eliminates this lag variance. Thus, with the chunk dejitter method, the large lag can easily be accounted for by subtracting the average lag from the EEG signal timestamps in a given run.

More important than this lag is the presence of timing jitter. This variation in timing precision is lowest for the Smarting Mobi (0.59 ms at 500 Hz and 1.17 ms at 250 Hz). Such variation is to be expected for these sampling frequencies. Overall, these results highlight the high and superior temporal precision of the Smarting Mobi amplifier. For the OpenBCI Cyton, the lowest jitter is found with the regular buffer setting and the chunk dejitter method (3.19 ms at 125 Hz and 1.69 ms at 250 Hz), closely followed by the recordings with the FTDI buffer fix (4.36 ms at 125 Hz and 2.10 ms at 250 Hz). These values imply that the OpenBCI Cyton+Daisy can be used reliably for the collection of ERPs with these configurations. The best performance is achieved with the regular buffer setting and the chunk dejitter method, which also showed very high consistency in jitter metrics (only minimally higher than for the Smarting Mobi). We, therefore, decided to use this configuration (default buffer setting + chunk dejitter timestamp correction) for the following amplifier comparisons with human participants.

## 4. Amplifier Comparison in Successive Recordings

After the timing test, we conducted an experiment with human participants to assess possible remaining differences in recording quality between the two amplifiers. Similar to previous amplifier comparison studies with traditional cap EEG [15,16], two paradigms were chosen to compare signal qualities, namely a comparison of frequency-domain features (band power differences due to visual stimulation and mental workload manipulation) and of time-domain features (the P300 ERP).

### 4.1. Protocol

In the first experimental stage, for the frequency-domain features, the Alpha band power differences between eyes-closed and eyes-open resting phases were compared (the Berger effect [10]). In these phases, participants were asked to sit still in front of a PC for 120 s with closed eyes or watching a light grey fixation cross on a dark grey screen. The magnitude of the band power differences is hereby considered the first measure of signal-to-noise ratio (SNR) for each amplifier. Similarly, Theta and Alpha band power differences were compared between low and high mental workload phases (again, for 120 s per phase—for a previous observation of the effect see [12,14]). For the induction of these workload levels, participants were asked to mentally sum a series of numbers. These additive equations were shown in a very simple form in the low workload condition (either “101 + 1 = ?”, “101 + 2 = ?”, or “101 + 3 = ?”), or in a hard form in the high workload condition (at least three double digit-summands, e.g., “68 + 71 + 31 = ?”). To ensure that the task was perceived as hard, the difficulty in the high workload condition was dynamically increased after each correctly solved trial by adding another summand or turning a single-digit summand into a double-digit summand. Participants had 18 s to complete a trial and enter the result in a field on the screen via a keyboard. Between each trial, a four second break occurred. The design for this task was chosen similar to the procedures used in studies that demonstrated successful elicitation of different mental workload levels [12,24,25]. Surveys were shown after each of these experimental tasks to assess perceived mental workload (using the NASA Task Load Index—TLX [26]) for a manipulation check.

In the second experiment stage, for the time-domain features, the P300 ERP was elicited by means of an auditory oddball task with 402 trials in total (divided into two blocks, with 80 standard and 20 target tones, and two blocks with 80 standard and 21 targets tones—block order was randomized to maintain participants’ concentration). The target tone had a frequency of 900 Hz the standard tone a frequency of 600 Hz. The order of tones was pseudo-randomized, and it was ensured that no more than two target tones were played in direct succession. A fixed inter-stimulus interval of 1000 ms was used. Participants were asked to count the occurrence of target tones in their head for each block while keeping their eyes open, and to write down their counts on a paper sheet next to them at the end of each block. Thereby, the procedure closely followed the one used in [10]. The stimuli were presented using NeuroBS Presentation. The amplitude of the ERP is herein considered the second SNR measure to evaluate the amplifiers’ recording qualities.

**Figure 8:**
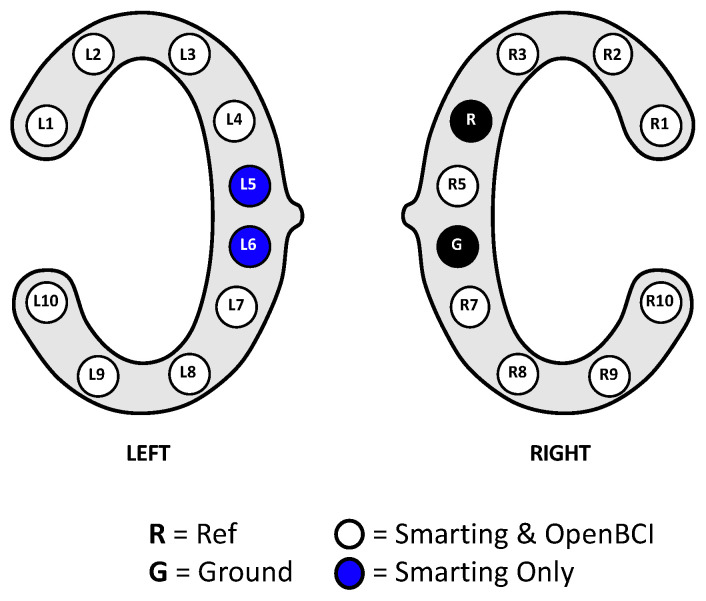
cEEGrid channel configurations for this first human participant study.

Stimulus presentation for the resting state and mental arithmetic task was performed in a self-developed Java program. All experiments were conducted on the same laptop running Windows 10, using the loudspeakers of the laptop to play the audio stimuli. LabStreamingLayer (LSL) was used to collect all the markers from the experimental software and the EEG amplifiers, thereby ensuring timestamp synchronization for all the data. For the Smarting Mobi recording, the adapter from mBrainTrain was used to connect the cEEGrids. Thereby, channel R4 served as the reference electrode (REF) and channel R6 as the ground (GND) electrode (see Figure 8). The same channels were chosen as REF and GND for the OpenBCI Cyton+Daisy amplifier. However, as the latter system only allows for an 18-channel recording, the L5 and L6 channels were left out of the recording. This decision was made to collect a symmetrical recording above and below the ear, especially for electrodes further away from the reference electrode—as the electrodes closest to the reference record the smallest amplitudes [3,10].

**Figure 9:**
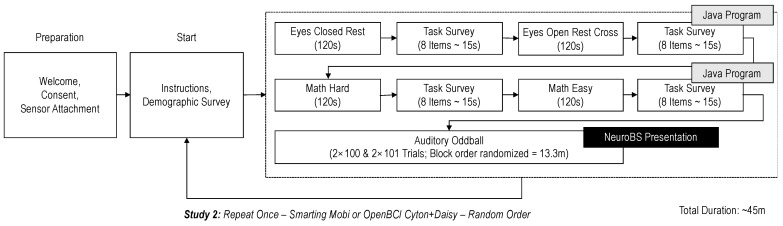
The experiment procedure visualized.

At the start of the experiment, participants were welcomed to the laboratory, received the participation instructions and signed the consent form. Before completing the main experiment tasks, participants also filled out a survey with demographic variables. To set up the recording, the participant’s skin was cleaned with alcohol and abrasive gel (Abralyt HiCl). A lentil-sized drop of gel was then placed on each cEEGrid electrode. An initial impedance check was performed, to ensure that the impedances on all channels were smaller than 30 kOhm before continuing the experiment. The overall procedure (including task orders and durations) is visualized in Figure 9. To assess the recording capabilities of each amplifier in their regular configuration, while minimizing the influence of inter-individual differences, each participant completed both experimental stages twice (i.e., in a within-subject design), once with the Smarting Mobi amplifier and once with the OpenBCI Cyton+Daisy amplifier. To eliminate the influence of order or duration effects on these results, the order in which amplifiers were used was randomized. Overall, 14 participants (six female, one left-handed) completed the study (seven participants for each amplifier order). All participants were generally healthy, had full vision (corrected or uncorrected) and participated voluntarily without receiving financial remuneration. The average age of participants is 25.3 years (median = 26, SD = 4.31).

### 4.2. Data Processing

Upon loading the EEG data, the streams were lag-corrected using the respective metrics determined in Section 3. The OpenBCI data was loaded with the “chunk dejitter” timestamp correction procedure. Then, the EEG data were processed following previous cEEGrid work, primarily the work by [10] to obtain comparable measures. This means that the channel data were mean-centered and re-referenced to a linked mastoid reference (mean of L4 and R4). To using ascertain a robust re-referencing, automated bad channel detection was performed using the Python version of the PREP pipeline [27]. Thereby, channels with minimal amplitudes (flat channels), abnormal deviations, and high degrees of high-frequency noise were removed and interpolated from the remaining electrodes. Only in one recording a single channel was removed and interpolated using this procedure. Further, during this early stage, the data of one participant had to be discarded due to large and persistent signal artifacts which were likely caused by electrode shift due to extensive sweating. Afterwards, the data were cut to extract and process the experimental conditions individually. A fully automated data preparation process (with descriptive statistical and visual inspection of the data before and after processing) was implemented to make the feature extraction transparent and reproducible (see, e.g., [27]). The signal processing and statistical analyses were conducted using custom Python scripts with the dedicated EEG Signal processing toolboxes (MNE Python—[28], megkit, PyPREP—[29]).

For the frequency band powers (for the resting and mental arithmetic conditions), 50 Hz line noise was removed using the ZapLine algorithm [30]. Afterwards, the data were bandpass filtered with a 2–15 Hz FIR filter to reduce the impact of broadband artefacts. These artefacts were prominent in the recordings with both amplifiers (see below as well) and have been previously documented [14]. Finally, to further reduce artefact influences, the signals were cleaned using Artefact Subspace Reconstruction (ASR—[31]) with the eyes open resting phase data as calibration data. Afterwards, band powers were extracted using the Welch periodogram (with 2 s window length—i.e., 1024 samples for the Smarting Mobi and 256 samples for the OpenBCI Cyton+Daisy and 50% window overlap with Hanning windowing). Frequency band powers were extracted for the Theta range (4–7 Hz) and Alpha range (8–12 Hz). To obtain more reliable frequency band estimates, the band powers were normalized by dividing each by the sum of all the frequency powers. Lastly, the powers were mean averaged across channels to obtain a single estimate and reduce the number of statistical tests.

For the ERP features, the signals were bandpass filtered first (0.2–15 Hz FIR filter). Afterwards the signals were visually inspected for artefact contamination. Unfortunately, numerous movement artefacts were discovered in many recordings. Therefore, ASR was used for these data as well (with 0.2–15 Hz filtered eyes-open resting state data as calibration data). Similar measures have been taken in related cEEGrid research where movement artefacts had to be removed [7]. Afterwards, as with the previous study, one-second-long epochs were extracted for each stimulus occurrence (−200 to +800 ms with 0 being the stimulus onset). All epochs were baseline corrected (subtracting the mean value of the segment from −200 ms to 0).

### 4.3. Results

#### 4.3.1. Frequency-Domain

For the frequency-domain amplifier comparison we replicated two known frequency band effects: (1) the Berger effect, and (2) the manipulation of frequency band powers by mental workload induction.

**Figure 10:**
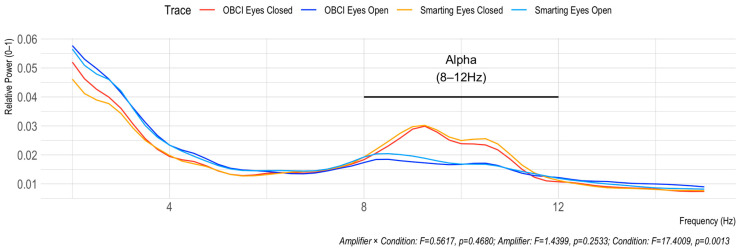
Berger effect replication. The elevated power in the Alpha frequency range (8–12 Hz) indicates similar effects for both amplifiers.

The Berger effect is the increase in Alpha frequency band power when the eyes are closed during rest. This effect is clearly visible by the peak in the Alpha range in Figure 10 for both amplifiers. Furthermore, a two-way repeated measures ANOVA (with interaction between Amplifier and Condition) shows a significant effect of Condition (F = 17.4009, *p* = 0.0013), but no effect of Amplifier (F = 1.4399, *p* = 0.2533) and no interaction effect (F = 0.5617, *p* = 0.4680). The absence of the interaction effect indicates that similar effects are observed for both amplifiers.

**Figure 11:**
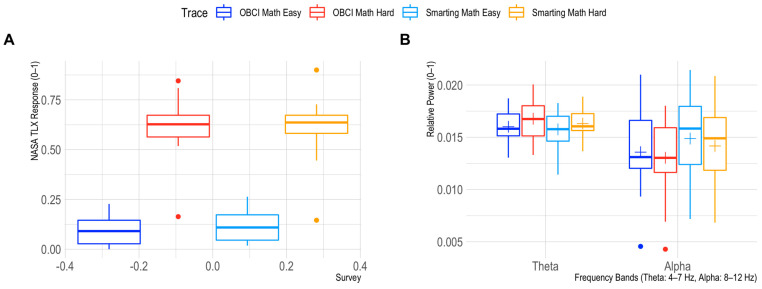
Mental workload effects of the easy and hard mental arithmetic task. Perceived differences are shown in (**A**), differences for the Theta and Alpha frequency bands for both amplifiers are shown in (**B**).

Next, to assess the effects of mental workload inductions on Theta and Alpha frequency band powers we first checked the manipulations’ success by comparing the perceived workload in the easy and hard mental arithmetic tasks. Therefore, the six NASA TLX report items were summed and normalized (range 0–1). This manipulation check shows a good separation of perceived mental workload between the task conditions (see Figure 11). For the frequency bands, we find a significant condition effect in the Theta range (F = 7.9723, *p* = 0.0154) and a trend level condition effect in the Alpha range (F = 3.9736, *p* = 0.0695). Additionally, the effects show the expected directions (increases for Theta and decreases for Alpha with higher load). For the Alpha band only, a significant amplifier effect is found (F = 7.2089, *p* = 0.0199) indicating higher Alpha powers overall for the Smarting Mobi recordings. As no interaction effects are found for either frequency band (Theta: F = 0.1890, *p* = 0.6715; Alpha: F = 0.1384, *p* = 0.7163), we conclude that also for this classical effect, no significant difference between the two amplifiers is found.

Altogether, this first set of results in the frequency-domain suggests that the Smarting Mobi and the OpenBCI Cyton+Daisy are similarly usable to study such neural activity. These observations are in line with previous work that observed similar effects with the OpenBCI Cyton+Daisy and cEEGrids [12]. However, as these are large-effect activities, this type of comparison only documents the lower capability bound. Additionally, it should be pointed out that oscillatory activity is less dependent on the temporal precision of the amplifiers. The more challenging comparison is, therefore, the following time-domain comparison of small-amplitude activities: ERPs.

#### 4.3.2. Time-Domain

The goal of this time-domain analysis was primarily to compare the SNR levels of the two amplifiers by replicating classical auditory evoked potentials. The amplitudes of these neural activities in the ear region (i.e., when recorded with cEEGrids) are typically very small due to the small inter-electrode distances (around ±3 µV—see e.g., [10]). Therefore, the observation of these ERPs can illuminate whether the timing and noise levels of the OpenBCI amplifier are good enough to compare to the Smarting Mobi for which the ERPs have been documented before [10]. In alignment with previous work [10], we pursued a qualitative and numerical comparison of the waveform morphologies, amplitudes, and condition effects.

**Figure 12:**
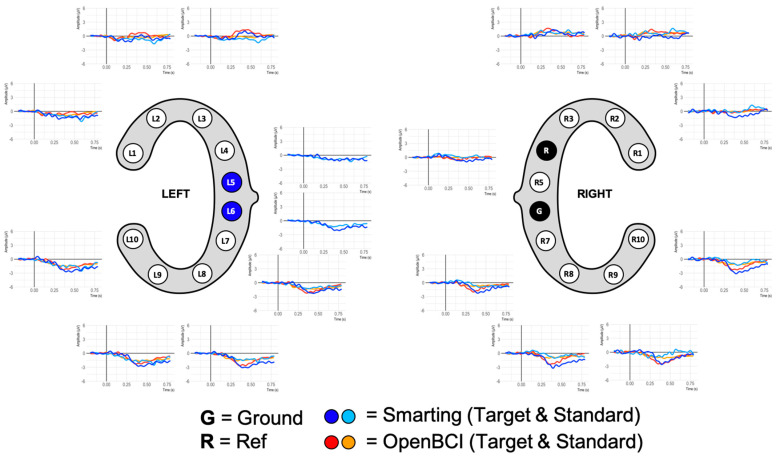
Grand average ERP plots for both amplifiers.

**Figure 13:**
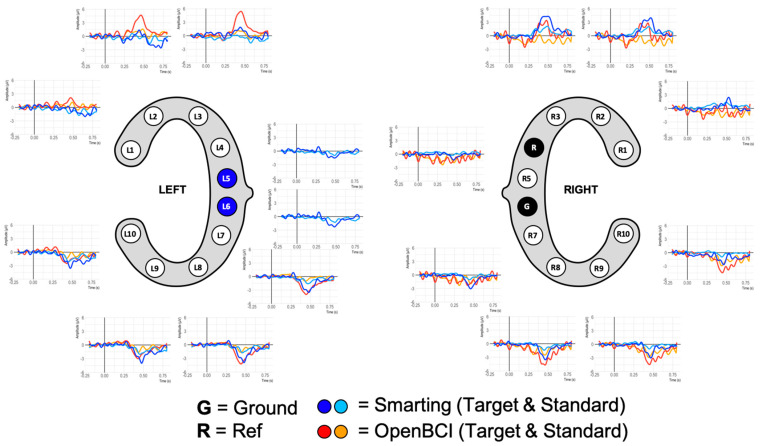
ERP Plots for a single participant with few recording artefacts.

The grand average ERPs are shown in Figure 12. While this figure shows ERP traces that can be described as expectable from this auditory attention task, the traces are more attenuated than in previous work for both amplifiers. The negative deflection approximately 100 ms after onset of both tones (N100) and the classical positive deflection after onset of the target tones (P300) are strongly attenuated. On the one hand, this is a normal effect due to the averaging of the results of multiple participants. Furthermore, the repetition of the experiment can have led to effect-diminishing habituation effects. On the other hand, the previously mentioned presence of movement artefacts is possibly also reducing the distinctive component morphology. By inspecting the data of one of the participants with the cleanest data (few movement artefacts) much larger N100 and P300 amplitudes can again be seen (see Figure 13). Both, the grand average and the single participant ERP traces also show the expected polarity reversal (a positive P300 deflection above and an inverted deflection below the L4+R4 reference) and show that these ERPs are more pronounced with greater distances to the reference. This component emphasis is most visible for the bottom electrodes (L8 and L9/R8/R9).

**Figure 14:**
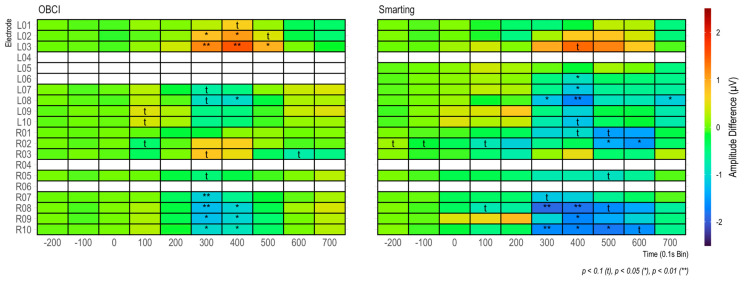
Amplitude differences in 100 ms bins for each amplifier. In addition, t-tests on amplitude differences are shown for each bin.

Furthermore, in both cases (grand average and individual participants) very similar amplitudes are observed for both amplifiers. Thus, from a qualitative view, we consider these ERP components to show high comparability of the two amplifiers in terms of SNR. To further assess the effect strength of the P300 component, amplitude differences between target and standard condition were calculated for 100 ms windows (see Figure 14). The condition effects are most pronounced between 300 and 500 ms and maximal at channels L8, L9, R8, and R9, with a much weaker, positive reflection of the same effect in the electrodes L2, L3, R2, and R3. To ascertain the significance of these differences for the entire sample, paired t-tests were calculated for each bin (see Figure 14). Both the heatmap and the statistical tests further support the observation of similar SNR across the amplifiers with the clearest effects for the evoked potential in the lower electrodes (L8 and L9/R8 and R9) in the 300 to 500 ms time range.

Altogether, through these various comparisons of the ERPs, the results suggest a similar aptitude of both amplifiers for recording ERPs with cEEGrids (i.e., low amplitude signals despite small electrode distances). However, the unexpected attenuation of the ERP traces led us to conduct a third recording and analysis aiming to provide an even more precise comparison of the time-domain effects by simultaneously recording cEEGrid data from a single participant in a single experiment run.

## 5. Amplifier Comparison in Concurrent Recording

The main motivation for this final study was the frequent presence of recording artefacts in the first user study and the general limitation from that repeated measures design that the observed signals might be subject to various behavioral and cognitive confounding factors (fatigue, boredom, or discomfort, just to name a few possible influences that might set in during the second repetition of the experiment). Therefore, to overcome these limitations and get a clear picture of the recording quality comparability of the two amplifiers with cEEGrids, a recording was conducted that concurrently used both amplifiers with a subset of the cEEGrid electrodes on each amplifier (as pairs with maximum proximity). Thereby, it was possible to measure a shared neural signal ground truth (similar to the timing test and similar to a previous OpenBCI comparison study [32]).

### 5.1. Protocol

**Figure 15:**
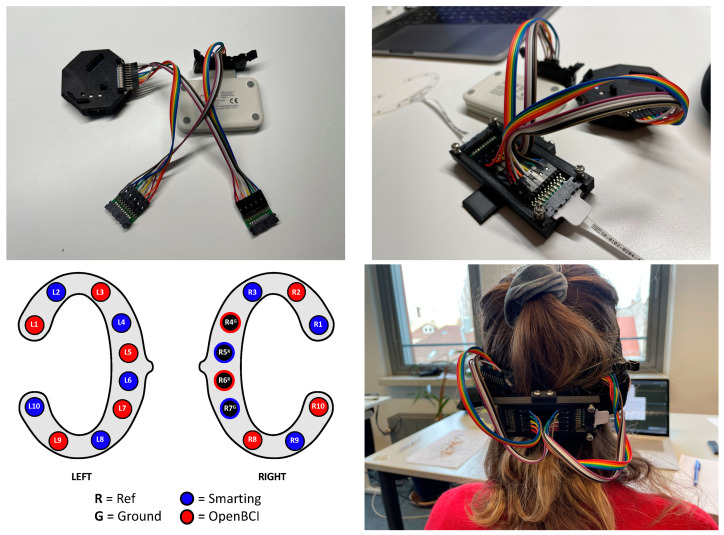
Channel and adapter configuration for a simultaneous recording with both amplifiers.

With the main interest focused on the time-domain signal comparability, the same experiment from the previous study (Section 4) was re-used, excluding the frequency-domain tasks (eyes-open and closed rest and mental arithmetic). Additionally, in this instance, the experiment was not repeated (single run). To simultaneously collect EEG data with both amplifiers, the cEEGrids were connected to a subset of the Smarting Mobi and the OpenBCI pins—in alternating order to assess the similarity of a simultaneous EEG recording on a single subject with electrodes in close proximity. The electrode configuration and a picture from the recording setup are shown in Figure 15. To obtain a higher sampling frequency (250 Hz) with the OpenBCI amplifier (and as only ten electrode positions are available for each amplifier), only the OpenBCI Cyton board (not the Daisy shield) were used.

This study was conducted with a single participant (female, age 25). The participant preparation and recording setup was completed in the same way as explained in Section 4.1. However, in this instance, after an initial impedance check to assure that all channels were available, we waited for 30 minutes for the impedances to settle (see, e.g., [33] for a demonstration of the impedance reduction in the first hour of an experiment with cEEGrids). The impedances in each channel were assessed using the Smarting Streamer Software (the standard adapter from mBrainTrain was used in this step). We also checked for good impedances in the reference and ground electrodes by rotating the connector. Afterwards, the impedances were recorded for two minutes before, and after the study recording to assess the possibility of signal quality changes over the course of the data collection (e.g., channel loss). Both before and after the experiment the impedances on all channels measured below 10 kOhm with no substantial changes during the experiment. Data were collected using the LabStreamingLayer (LSL) protocol for precise integration of amplifier and experiment timestamps. The sampling frequencies were both set to be as high as possible (500 Hz for Smarting Mobi, 250 Hz for OpenBCI) to allow a comparison with both amplifiers performing at their highest level.

### 5.2. Data Processing

The OpenBCI data was again loaded with the “chunk dejitter” timestamp correction procedure documented in the previous chapters. The lags for Smarting and OpenBCI were subtracted from each amplifiers’ timestamps to align the signals. Again, as in Section 4.1 the recorded signals were band-pass filtered (0.2–15 Hz, FIR) for the following analyses. Afterwards, trial epochs were extracted over the range of −0.2 to 0.8 s around stimulus onset. The average amplitude from −0.2 s before onset to 0 s was subtracted from each epoch’s signal to baseline-correct each trial’s data.

### 5.3. Results

**Figure 16:**
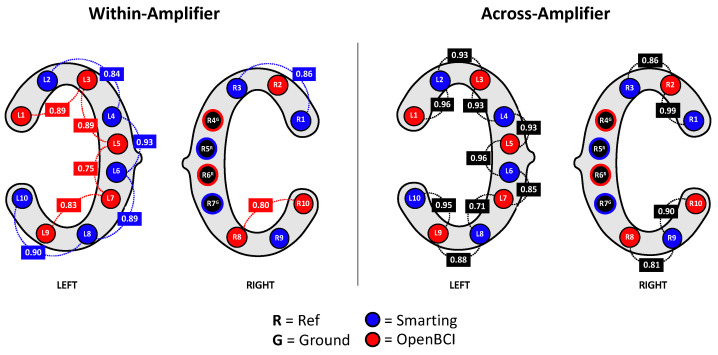
Correlations between channels within amplifiers (**left**) and across amplifiers (**right**).

**Figure 17:**
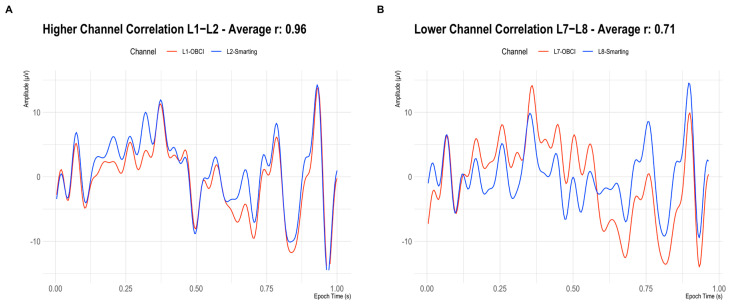
Signals on two adjacent electrodes (Smarting Mobi = Blue, OpenBCI Cyton = Red) in a highly correlated pair (**A**) and a less correlated pair (**B**).

Besides comparing the ERP components as in the previous study (Section 4), we decided to also directly compare the recorded signals’ similarity across amplifiers by inspecting the signal correlation in adjacent electrodes. First, as a reference point, the correlations for adjacent electrodes for each individual amplifier were calculated. These within-amplifier channel correlations showed similarly high coefficients for both amplifiers (Smarting Mobi: 0.89, OpenBCI: 0.83). Figure 16 shows the full distribution of channel correlations. Next, for the channel correlation assessment across amplifiers, the Smarting Mobi data was downsampled to 250 Hz. The median channel correlation with mixed-amplifier pairs was found to be similarly high, albeit slightly higher with r = 0.93. These higher cross-amplifier correlations are most likely caused by the smaller electrode distances. Importantly, this finding indicates highly comparable SNR in electrodes with close proximity across amplifiers. Figure 17 shows two channels in close proximity with higher and lower correlations.

**Figure 18:**
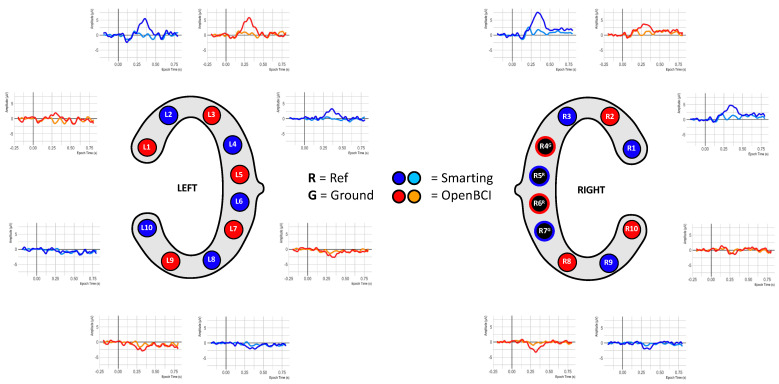
ERP signals from simultaneous recordings.

Given this high signal comparability, the final ERP comparison was pursued. To enable higher comparability, a crossed linked reference was computed (i.e., L6+R5 for the Smarting Mobi, and L5+R6 for the OpenBCI Cyton). Next, all trials per condition (standard and target) were averaged. Figure 18 shows the ERP traces aligned with the cEEGrid electrodes.

This figure shows ERP traces that can be described as to be expected from this auditory attention task. A negative deflection approximately 100 ms after onset of both tones is visible, resembling an auditory evoked potential N100 component (see, e.g., [34]). This deflection is pronounced in the upper channels (L1 to L3 and R1 to R3) and is diminished or absent in the other channels. Furthermore, an even more prominent positive deflection in response to target tones is found, with peak amplitudes at approximately 400 ms. Morphology, condition effect and latency of this deflection strongly resembled the typical P300 ERP component (see, e.g., [10,35]). In a similar manner to the N100, the P300 was pronounced for channels located above the reference sites. Below the references an opposite polarity waveform emerges with a smaller amplitude. Comparing the traces across amplifiers, the signals are very similar (see, e.g., L2 and L3 for two very similar traces in adjacent electrodes—or L3 and R3 for two very similar traces on two contralateral electrodes). However, slight differences remain that could be due to the differences resulting from the reference electrode positions or anatomical peculiarities.

**Figure 19:**
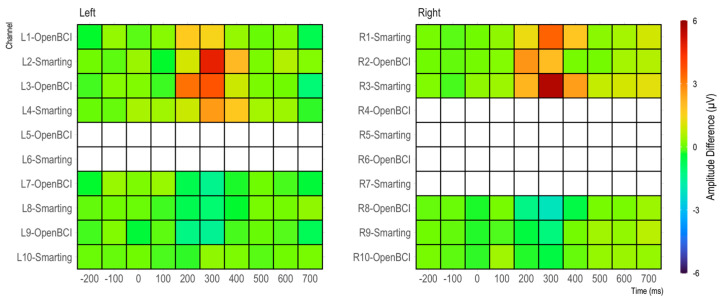
Amplitude differences by channel in 100 ms bins.

As in the previous study, to further assess the effect strength of the P300 component, differences between target and standard condition were calculated for 100 ms windows (see Figure 19). Again, the condition effects are most pronounced between 300 and 500 ms and maximal at channels L2 and L3/R3 and R3—with a weaker, negative reflection of the same effect in the electrodes L8 and L9/R8 and R9. From this comparison as well, no clear difference in terms of effect strength emerges between the two amplifiers.

In summary, this final, concurrent time-domain comparison of the two amplifiers again supports the observation that similar cEEGrid recording aptitudes are found for both the Smarting Mobi and the OpenBCI Cyton amplifier.

## 6. Discussion and Conclusions

In this work, we compared the performance of a high-end (MBrainTrain Smarting Mobi) and a low-cost (OpenBCI Cyton+Daisy) EEG signal amplifier for recording neural activity with around-the-ear (cEEGrid) electrodes. This comparison provides crucial answers for the accessibility and progress of the research on concealed EEG, which can be used in everyday life to enable applications such as adaptive hearing aids [3], sleep monitoring [1] or novel human–computer interaction modalities [4,5]. Due to the high technical requirements for the recording of ear EEG signals (high temporal precision for ERPs and high SNR for the acquisition of low-amplitude signals [3]) a comprehensive evaluation of the recording capabilities of a low-cost amplifier alternative is essential. Thereby, our work goes beyond previous comparisons of the OpenBCI Cyton+Daisy amplifier [15,32] by documenting temporal precision more systematically and directly comparing ear-EEG signals and features. We adapted a paradigm specifically for this purpose and identified a number of interesting parameters.

First of all, our work shows that it is necessary to determine the desired recording configuration with the OpenBCI Cyton+Daisy and the USB dongle buffer settings. If the system is used with its default settings (“out-of-the-box”), scholars will experience problems with latency and jitter—especially when using the currently available dejittering methods (i.e., those implemented in the XDF interfaces like pyxdf). While the OpenBCI developers are aware of this limitation and recommend the FTDI buffer fix in the amplifier documentation, this step may not be obvious to all users. For example, we were not aware of this aspect when recording the timing test signals. On the other hand, this led us to investigate and develop a fix for the chunky and irregular timestamp patterns in this initial configuration—the chunk dejitter method. This new algorithm not only allowed for the correction of misaligned timestamps, but actually led to improved timing performance (higher precision and consistency), which is why we used (and recommend) it for our ERP study recordings. The improved consistency is the main reason for this recommendation, as the OpenBCI Cyton+Daisy sometimes showed quite high jitter variances. Even with the manufacturer’s recommended FTDI buffer fix, sample transfer timestamps can be quite volatile, which can have a serious impact on ERP analyses. Still, we must emphasize that our recommended recording configuration without the buffer fix will require a one-time shift of the recorded timestamps to provide accurate ERP results due to the high lag of over 500 ms. In summary, with these recommended corrections (using the new chunk dejitter and timestamp shift) OpenBCI Cyton+Daisy users can reliably (i.e., with high temporal precision) and easily (without having to change hardware settings) record ear-EEG time-domain signals that approach the quality of the high-end Smarting Mobi amplifier.

Second, beyond these timing findings, we found comparable performance in human cEEGrid recordings with both amplifiers in two experimental designs: (1) repeated experiments with both amplifiers per participant, and (2) simultaneous amplifier recordings in a single experiment run. Generally, it should be highlighted that our results rely on a relatively small sample size, which poses a limitation for the detection of small effects. However, as we were primarily interested in replicating relatively large and well-known effects—and did so with both amplifiers—we feel that this sample size was acceptable for the purposes of this work. In the first case, comparable signals are found for the frequency-domain and time-domain features, with no discernible patterns that would indicate superior performance of one amplifier over the other. However, since the recorded data in this first experiment showed general limitations with artifacts, we also looked more closely at the concurrent recordings. In this last experiment, we were able to directly assess signal comparability and found very similar signal morphologies and ERP components for both amplifiers. Again, these results lead us to conclude that both amplifiers have similar SNR performance. Because the signals were compared for electrodes in close proximity, but not exactly in the same position, there remain minimal differences that could be further eliminated by building adapters that allow signals from the same electrodes to be recorded simultaneously with both amplifiers. A design for such an adapter (which also limits crosstalk between amplifiers connected to the same electrode) has been documented in [32]. However, since the author found very similar signals in the OpenBCI Cyton and another high-end EEG amplifier, and since our results have consistently shown similar signal qualities, we believe that the sum of the evidence sufficiently documents the suitability of the OpenBCI Cyton+Daisy amplifier for recording (around-the-ear) EEG signals.

In conclusion, the MBrainTrain Smarting Mobi definitely offers advantages in terms of sampling frequency and temporal precision for the study of neural signals in the ear region. If money is no obstacle, it is probably the preferably option currently. However, if you are on a smaller budget, using the OpenBCI Cyton+Daisy amplifiers is a viable alternative for (around-the-ear) EEG research and prototype development. Given that many of these amplifiers are already distributed around the world (OpenBCI currently lists over 200 publications with their devices, which is only a fraction of the units distributed) this new evidence of additional recording capabilities will hopefully stimulate further growth in the development of concealed EEG [3], which has the potential to deliver exciting applications and technologies in the near future.

## Figures and Tables

**Table 1:** Amplifier specifications in comparison.

Characteristic	OpenBCI Cyton	OpenBCI Cyton+Daisy	Smarting Mobi 24
**Recording Channels** **(excluding REF and GND)**	8	16	22
**Sampling Frequency**	250 Hz	125 Hz	500 or 250 Hz
**SD Card Recording**	Yes		No
**Data Transmission**	RFDuino Bluetooth Dongle—4.0 Low Energy (BLE) radio transceiver		BlueSoleil Bluetooth Dongle Class I with Bluetooth v2.1 + EDR
**Recording Duration (Power)**	>12 h		~4 h
**Configurability**	All Hardware and Software		API for online data processing
**REF/GND Configuration**	Passive		CMS/DRL
**Input-referred noise**	~1 µVpp		<1 µVpp
**Com. Mode Rejection Ratio**	~110 dB		>110 dB
**Max. Amplification**	×24		
**Resolution**	24 bit		
**Movement Sensor**	three-axis accelerometers		

## Data Availability

The timing test data and analysis code (Section 3) can be found with this repository: https://github.com/MKnierim/openbci-vs-smarting-timing-test; The data from the human participant recordings are not publicly available due to their sensitive nature.

## Supplementary Materials

The following supporting information can be downloaded at: https://github.com/MKnierim/pyxdf (accessed on 3 May 2023), The chunk dejitter algorithm implemented as an extension of the PyXDF library.

## References

[1] Choi J., Kwon M., Jun S.C. (2020). A systematic review of closed-loop feedback techniques in sleep studies—Related issues and future directions. Sensors.

[2] Gu Y., Cleeren E., Dan J., Claes K., Van Paesschen W., Van Huffel S., Hunyadi B. (2017). Comparison between scalp EEG and behind-the-ear EEG for development of a wearable seizure detection system for patients with focal epilepsy. Sensors.

[3] Bleichner M.G., Debener S. (2017). Concealed, unobtrusive ear-centered EEG acquisition: Ceegrids for transparent EEG. Front. Hum. Neurosci..

[4] Rezeika A., Benda M., Stawicki P., Gembler F., Saboor A., Volosyak I. (2018). Brain–computer interface spellers: A review. Brain Sci..

[5] Blankertz B., Acqualagna L., Dähne S., Haufe S., Schultze-Kraft M., Sturm I., Ušcumlic M., Wenzel M.A., Curio G., Müller K.-R. (2016). The Berlin Brain-Computer Interface: Progress Beyond Communication and Control. Front. Neurosci..

[6] Meiser A., Bleichner M.G. (2022). Ear-EEG compares well to cap-EEG in recording auditory ERPs: A quantification of signal loss. J. Neural Eng..

[7] Hölle D., Meekes J., Bleichner M.G. (2021). Mobile ear-EEG to study auditory attention in everyday life. Behav. Res. Methods.

[8] Hölle D., Blum S., Kissner S., Debener S., Bleichner M.G. (2022). Real-Time Audio Processing of Real-Life Soundscapes for EEG Analysis: ERPs Based on Natural Sound Onsets. Front. Neuroergon..

[9] Valentin O., Viallet G., Delnavaz A., Cretot-Richert G., Ducharme M., Monsarat-Chanon H., Voix J. (2021). Custom-fitted in-and around-the-ear sensors for unobtrusive and on-the-go eeg acquisitions: Development and validation. Sensors.

[10] Debener S., Emkes R., De Vos M., Bleichner M. (2015). Unobtrusive ambulatory EEG using a smartphone and flexible printed electrodes around the ear. Sci. Rep..

[11] Hölle D., Bleichner M.G. (2023). Smartphone-based ear-EEG to study sound processing in everyday life. bioRxiv.

[12] Knierim M.T., Berger C., Reali P. (2021). Open-Source Concealed EEG Data Collection for Brain-Computer-Interfaces: Neural Observation Through OpenBCI Amplifiers with Around-the-Ear cEEGrid Electrodes. Brain-Comput. Interfaces.

[13] Berger H. (1929). Über das Elektroenkephalogramm des Menschen. Arch. Psychiatr. Nervenkr..

[14] Wascher E., Arnau S., Reiser J.E., Rudinger G., Karthaus M., Rinkenauer G., Dreger F., Getzmann S. (2019). Evaluating Mental Load During Realistic Driving Simulations by Means of Round the Ear Electrodes. Front. Neurosci..

[15] Rashid U., Niazi I.K., Signal N., Taylor D. (2018). An EEG experimental study evaluating the performance of Texas instruments ADS1299. Sensors.

[16] Frey J. Comparison of an open-hardware electroencephalography amplifier with medical grade device in brain-computer interface applications. Proceedings of the PhyCS 2016—Proceedings of the 3rd International Conference on Physiological Computing Systems.

[17] Goverdovsky V., Von Rosenberg W., Nakamura T., Looney D., Sharp D.J., Papavassiliou C., Morrell M.J., Mandic D.P. (2017). Hearables: Multimodal physiological in-ear sensing. Sci. Rep..

[18] Mikkelsen K.B., Kappel S.L., Mandic D.P., Kidmose P. (2015). EEG recorded from the ear: Characterizing the Ear-EEG Method. Front. Neurosci..

[19] Kappel S.L., Makeig S., Kidmose P. (2019). Ear-EEG Forward Models: Improved Head-Models for Ear-EEG. Front. Neurosci..

[20] Meiser A., Tadel F., Debener S., Bleichner M.G. (2020). The Sensitivity of Ear-EEG: Evaluating the Source-Sensor Relationship Using Forward Modeling. Brain Topogr..

[21] Sawangjai P., Hompoonsup S., Leelaarporn P., Kongwudhikunakorn S., Wilaiprasitporn T. (2019). Consumer Grade EEG Measuring Sensors as Research Tools: A Review. IEEE Sens. J..

[22] Hölle D., Bleichner M.G. (2022). Recording brain activity with ear-EEG (cEEGrids). bioRxiv.

[23] Blum S., Debener S., Emkes R., Volkening N., Fudickar S., Bleichner M.G. (2017). EEG Recording and Online Signal Processing on Android: A Multiapp Framework for Brain-Computer Interfaces on Smartphone. Biomed Res. Int..

[24] Ulrich M., Keller J., Hoenig K., Waller C., Grön G. (2014). Neural correlates of experimentally induced flow experiences. Neuroimage.

[25] Katahira K., Yamazaki Y., Yamaoka C., Ozaki H., Nakagawa S., Nagata N. (2018). EEG correlates of the flow state: A combination of increased frontal theta and moderate frontocentral alpha rhythm in the mental arithmetic task. Front. Psychol..

[26] Hart S.G., Staveland L.E. (1988). Development of NASA-TLX (Task Load Index): Results of empirical and theoretical research. Adv. Psychol..

[27] Bigdely-Shamlo N., Mullen T., Kothe C., Su K.-M., Robbins K.A. (2015). The PREP pipeline: Standardized preprocessing for large-scale EEG analysis. Front. Neuroinform..

[28] Gramfort A., Luessi M., Larson E., Engemann D.A., Strohmeier D., Brodbeck C., Goj R., Jas M., Brooks T., Parkkonen L. (2013). MEG and EEG data analysis with MNE-Python. Front. Neurosci..

[29] Appelhoff S., Hurst A.J., Lawrence A., Li A., Mantilla Ramos Y.J., O’Reilly C., Xiang L., Dancker J. (2022). PyPREP: A Python Implementation of the Preprocessing Pipeline (PREP) for EEG Data. https://zenodo.org/record/6363576#.ZFUi--xBy3I.

[30] de Cheveigné A. (2020). ZapLine: A simple and effective method to remove power line artifacts. Neuroimage.

[31] Mullen T.R., Kothe C.A.E., Chi M., Ojeda A., Kerth T., Makeig S., Jung T.-P., Cauwenberghs G. (2015). Real-time Neuroimaging and Cognitive Monitoring Using Wearable Dry EEG. IEEE Trans. Biomed. Eng..

[32] Frey J. Comparison of a consumer grade EEG amplifier with medical grade equipment in BCI applications. Proceedings of the International BCI Meeting.

[33] Knierim M.T., Schemmer M., Bauer N. (2022). A Simplified Design of a cEEGrid Ear-Electrode Adapter for the OpenBCI Biosensing Platform. HardwareX.

[34] Hine J., Debener S. (2007). Late auditory evoked potentials asymmetry revisited. Clin. Neurophysiol..

[35] Polich J. (2007). Updating P300: An integrative theory of P3a and P3b. Clin. Neurophysiol..

